# Anomalous critical fields in quantum critical superconductors

**DOI:** 10.1038/ncomms6679

**Published:** 2014-12-05

**Authors:** C. Putzke, P. Walmsley, J. D. Fletcher, L. Malone, D. Vignolles, C. Proust, S. Badoux, P. See, H. E. Beere, D. A. Ritchie, S. Kasahara, Y. Mizukami, T. Shibauchi, Y. Matsuda, A. Carrington

**Affiliations:** 1 H. H. Wills Physics Laboratory, University of Bristol, Tyndall Avenue, Bristol BS8 1TL, UK; 2 National Physical Laboratory, Hampton Road, Teddington TW11 0LW, UK; 3 Laboratoire National des Champs Magnétiques Intenses (CNRS-INSA-UJF-UPS), 31400 Toulouse, France; 4 Cavendish Laboratory, University of Cambridge, J.J. Thomson Avenue, Cambridge CB3 0HE, UK; 5 Department of Physics, Kyoto University, Sakyo-ku, Kyoto 606-8502, Japan; 6 Department of Advanced Materials Science, University of Tokyo, Kashiwa 277-8561, Japan

## Abstract

Fluctuations around an antiferromagnetic quantum critical point (QCP) are believed to lead to unconventional superconductivity and in some cases to high-temperature superconductivity. However, the exact mechanism by which this occurs remains poorly understood. The iron-pnictide superconductor BaFe_2_(As_1−*x*_P_*x*_)_2_ is perhaps the clearest example to date of a high-temperature quantum critical superconductor, and so it is a particularly suitable system to study how the quantum critical fluctuations affect the superconducting state. Here we show that the proximity of the QCP yields unexpected anomalies in the superconducting critical fields. We find that both the lower and upper critical fields do not follow the behaviour, predicted by conventional theory, resulting from the observed mass enhancement near the QCP. Our results imply that the energy of superconducting vortices is enhanced, possibly due to a microscopic mixing of antiferromagnetism and superconductivity, suggesting that a highly unusual vortex state is realized in quantum critical superconductors.

## Abstract (web summary)

Superconductivity in the iron pnictides is believed to be related to quantum critical fluctuations. Putzke *et al.* observe unexpected anomalies in the critical fields of BaFe_2_(As_1−x_P_x_)_2_ that emerge close to its magnetic critical point, which they argue is a generic feature of quantum critical superconductivity.

## Graphical abstract

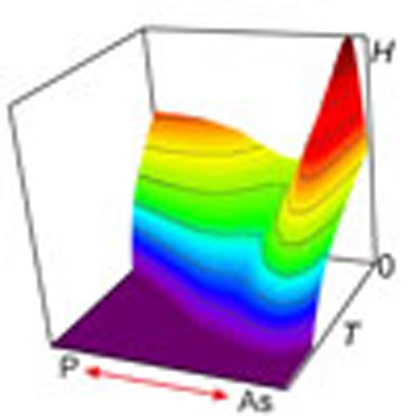

Quantum critical points (QCPs) can be associated with a variety of different order–disorder phenomena, however, so far superconductivity has only been found close to magnetic order. Superconductivity in heavy fermions, iron pnictides and organic salts is found in close proximity to antiferromagnetic order^1^,^2^, whereas in the cuprates the nature of the order (known as the pseudogap phase) is less clear^3^. The normal state of these materials has been widely studied and close to their QCPs non-Fermi liquid behaviour of transport and thermodynamic properties are often found, however, comparatively little is known about how the quantum critical fluctuations affect the superconducting state^4^. This is important as it is the difference in energy between the normal and superconducting state that ultimately determines the critical temperature *T*_c_.

Among the various iron-pnictide superconductors, BaFe_2_(As_1−*x*_P_*x*_)_2_ has proved to be the most suitable family for studying the influence of quantum criticality on the superconducting state. This is because the substitution of As by P introduces minimal disorder as it tunes the material across the phase diagram from a spin-density wave antiferromagnetic metal, through the superconducting phase to a paramagnetic metal^5^. The main effect is a compression of the *c* axis arising from the smaller size of the P ion compared with As, which mimics the effect of external pressure^6^. Normal state properties such as the temperature dependence of the resistivity^7^ and spin-lattice relaxation rate^8^ clearly point to a QCP at *x*=0.30. Measurements of superconducting state properties that show signatures of quantum critical effects include the magnetic penetration depth *λ* and the heat capacity jump at *T*_c_, Δ*C*^9^,^10^. Both of these quantities show a strong increase as *x* tends to 0.30, and it is shown that this could be explained by an underlying approximately sixfold increase in the quasiparticle effective mass m* at the QCP^10^.

In the standard single-band Ginzburg–Landau theory, the upper critical field is given by


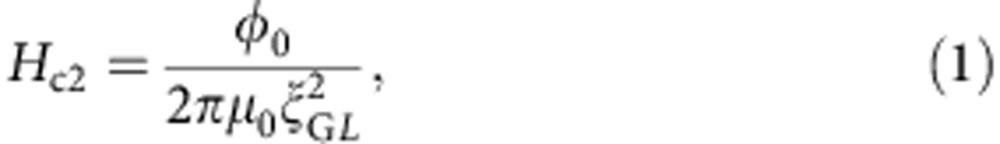


where *φ*_0_ is the flux quantum and *ξ*_GL_ is the Ginzburg–Landau coherence length. In the clean limit at low temperature, *ξ*_GL_ is usually well approximated by the BCS coherence length, which results in *H*_c2_∝(m*Δ)^2^, where m* is the mass of the quasiparticles and Δ is the superconducting gap. This simplified analysis is borne out by the full strong coupling BCS theory^11^. Hence, a strong peak in m* at the QCP should result in a corresponding increase in *H*_c2_ as well as the slope of *H*_c2_ at 
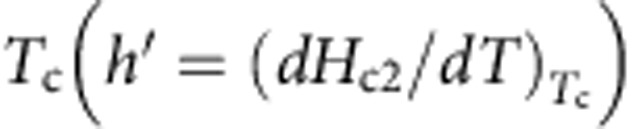
. This latter quantity is often more easily accessible experimentally because of the very high *H*_c2_ values in compounds such as iron pnictides for *T*≪*T*_c_ and also because the values of *H*_c2_ close to *T*_c_ are not reduced by the effect of the magnetic field on the electron spin (Pauli limiting effects).

For the lower critical field *H*_c1_, standard Ginzburg–Landau theory predicts that


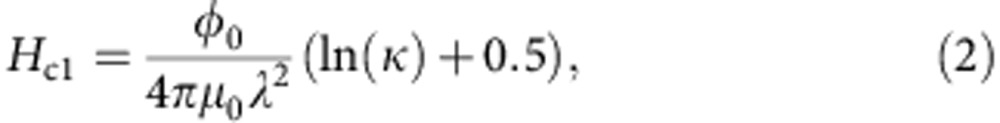


where *κ*=*λ*/*ξ*_GL_, and so the observed large peak in *λ* at the QCP^9^ should result in a strong suppression of *H*_c1_. Here we show that the exact opposite, a peak in *H*_c1_ at the QCP, occurs in BaFe_2_(As_1−*x*_P_*x*_)_2_, and in addition the expected sharp increase in *H*_c2_ is not observed. This suggests that the critical fields of quantum critical superconductors strongly violate the standard theory.

## Results

### Upper critical field *H*_c2_

**Figure 1:**
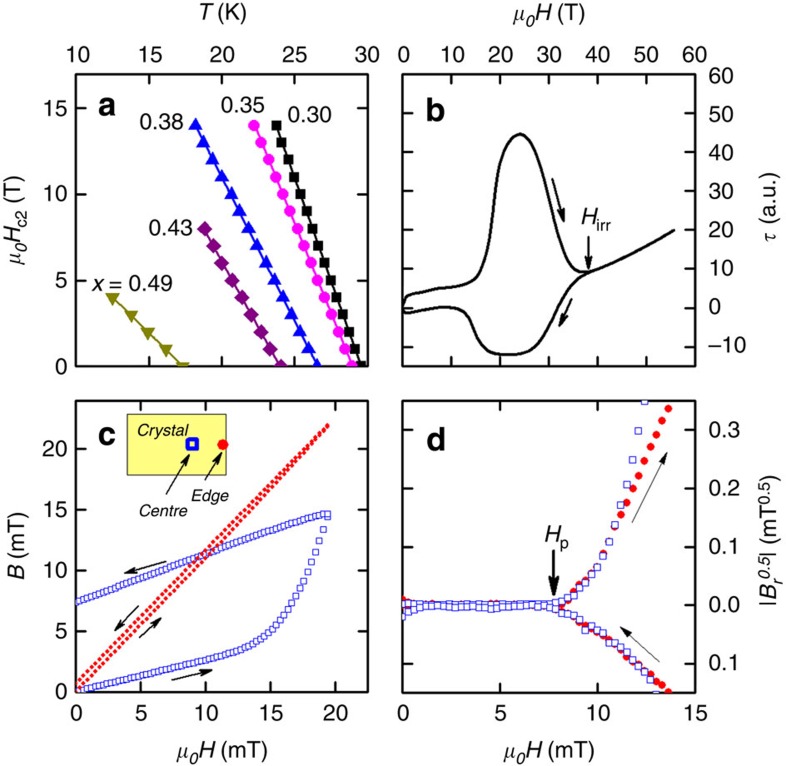
Determination of critical fields. (**a**) *H*_c2_(*T*) data close to *T*_c_(*H*=0) from heat capacity measurements for different samples of BaFe_2_(As_1−*x*_P_*x*_)_2_. (**b**) Magnetic torque versus rising and falling field for a sample with *x*=0.40 at *T*=1.5 K. The irreversibility field *H*_irr_ is marked. (**c**) Magnetic flux density *B* versus applied field *H* as measured by the micro-Hall sensors, for *x*=0.35 and *T*=18 K at two different sensor positions: one at the edge of the sample and the other close to the centre (schematic inset). (**d**) Remnant field *B*_r_ after subtraction of the linear term due to flux leakage around the sample. |*B*_r_|^0.5^ versus *μ*_0_*H* is plotted as this best linearizes *B*_r_(*H*)^14^. Note that the changes in linearity of *B*(*H*) evident in **d** are not visible by eye in **c**.

**Figure 2:**
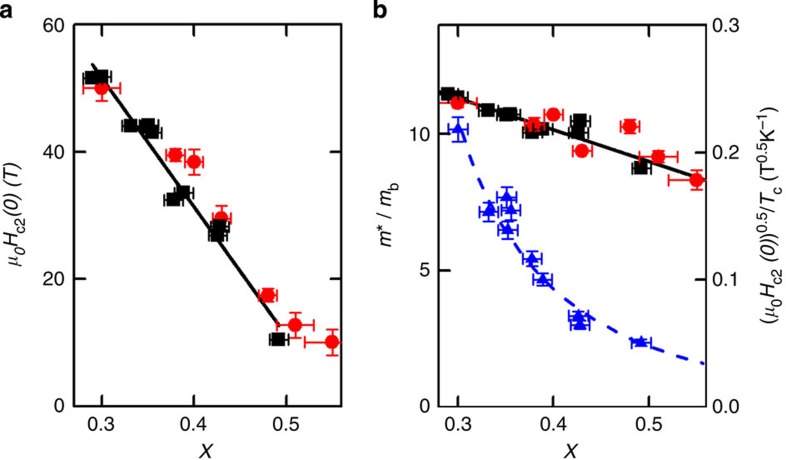
Upper critical field as a function of concentration *x*. (**a**) *H*_c2_(0) in BaFe_2_(As_1−*x*_P_*x*_)_2_ estimated from the slope of *H*_c2_(*T*) close to *T*_c_ using 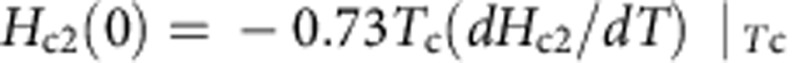 (squares) 13, and also estimates of *H*_c2_(0) from the irreversibility field at low temperature (*T*=1.5 K) measured by torque magnetometry (circles). Error bars on *H*_c2_ (circles) represent the uncertainties in locating *H*_irr_ and (squares) in extrapolating the values close to *T*_c_ to *T*=0. Error bars on *x* represent s.d. (**b**) The same data plotted as (*H*_c2_(0))^0.5^/*T*_c_, which, in conventional theory, are proportional to the mass enhancement m*. The mass renormalization m*/*m*_b_ derived from specific heat measurements is shown for comparison (triangles) 10. The dashed line is a guide to the eye and solid lines in both parts are linear fits to the data.

We measured *H*_c2_ parallel to the *c* axis, in a series of high-quality single-crystal samples of BaFe_2_(As_1−*x*_P_*x*_)_2_ spanning the superconducting part of the phase diagram using two different techniques. Close to *T*_c_(*H*=0), we measured the heat capacity of the sample using a microcalorimeter in fields up to 14 T (see Fig. 1a). This gives an unambiguous measurement of *H*_c2_(*T*) and the slope *h*′, which unlike transport measurements is not complicated by contributions from vortex motion^12^. At a lower temperature, we used micro-cantilever torque measurements in pulsed magnetic fields up to 60 T. Here an estimate of *H*_c2_ was made by observing the field where hysteresis in the torque magnetization loop closes (see Fig. 1b). Although, strictly speaking, this marks the irreversibility line *H*_irr_, this is a lower limit for *H*_c2_(0) and in superconductors with negligible thermal fluctuations and low anisotropy such as BaFe_2_(As_1−*x*_P_*x*_)_2_ *H*_irr_ should coincide approximately with *H*_c2_. Indeed, in Fig. 2 we show that the extrapolation of the high-temperature-specific heat results, using the Helfand–Werthamer (HW) formula^13^, to zero temperature are in good agreement with the irreversibility field measurements showing both are good estimates of *H*_c2_(0).

In the clean limit we would expect (*H*_c2_(0))^1/2^/*T*_c_ to be proportional to the renormalized effective mass m*. Surprisingly, we show in Fig. 2 that this quantity increases by just ~20% from *x*=0.47 to *x*=0.30, whereas m* increases by ~400% for the same range of *x*.

### Lower critical field *H*_c1_

We measured *H*_c1_ in our BaFe_2_(As_1−*x*_P_*x*_)_2_ samples using a micro-Hall probe array. Here the magnetic flux density *B* is measured at several discrete points a few microns from the surface of the sample. Below *H*_c1_, *B* increases linearly with the applied field *H* due to incomplete shielding of the sensor by the sample. Then, as the applied field passes a certain field *H*_p_, *B* increases more rapidly with *H* indicating that vortices have entered the sample (see Fig. 1c,d). Care must be taken in identifying *H*_p_ with *H*_c1_ because, in some cases, surface pinning and geometrical barriers can push *H*_*p*_ well above *H*_c1_. However, in our measurements, several different checks, such as the equality of *H*_*p*_ for increasing and decreasing field^14^, and the independence of *H*_p_ on the sensor position^15^, rule this out (see Methods).

**Figure 3:**
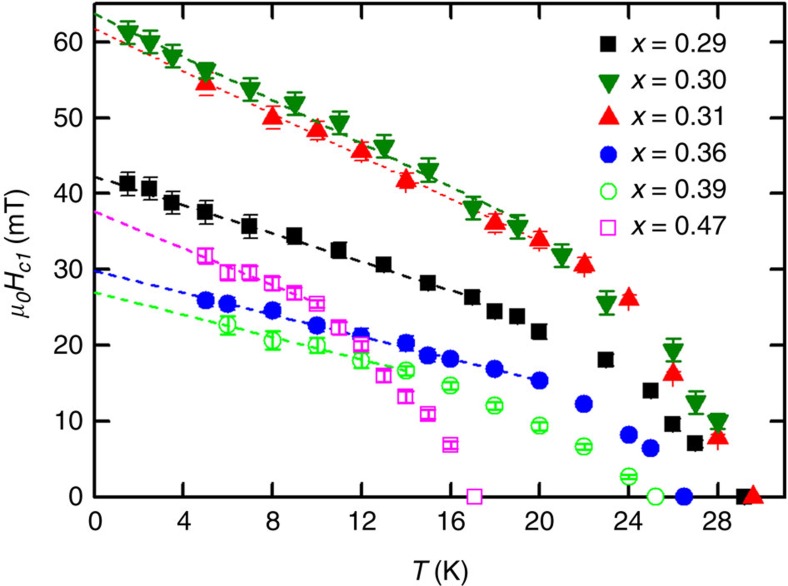
Temperature dependence of *H*_c1_ in samples of BaFe_2_(As_1−*x*_P_*x*_)_2_. The lines show the linear extrapolation used to determine the value at *T*=0. Error bars represent the uncertainty in locating *H*_c1_ from the raw *B*(*H*) data.

**Figure 4:**
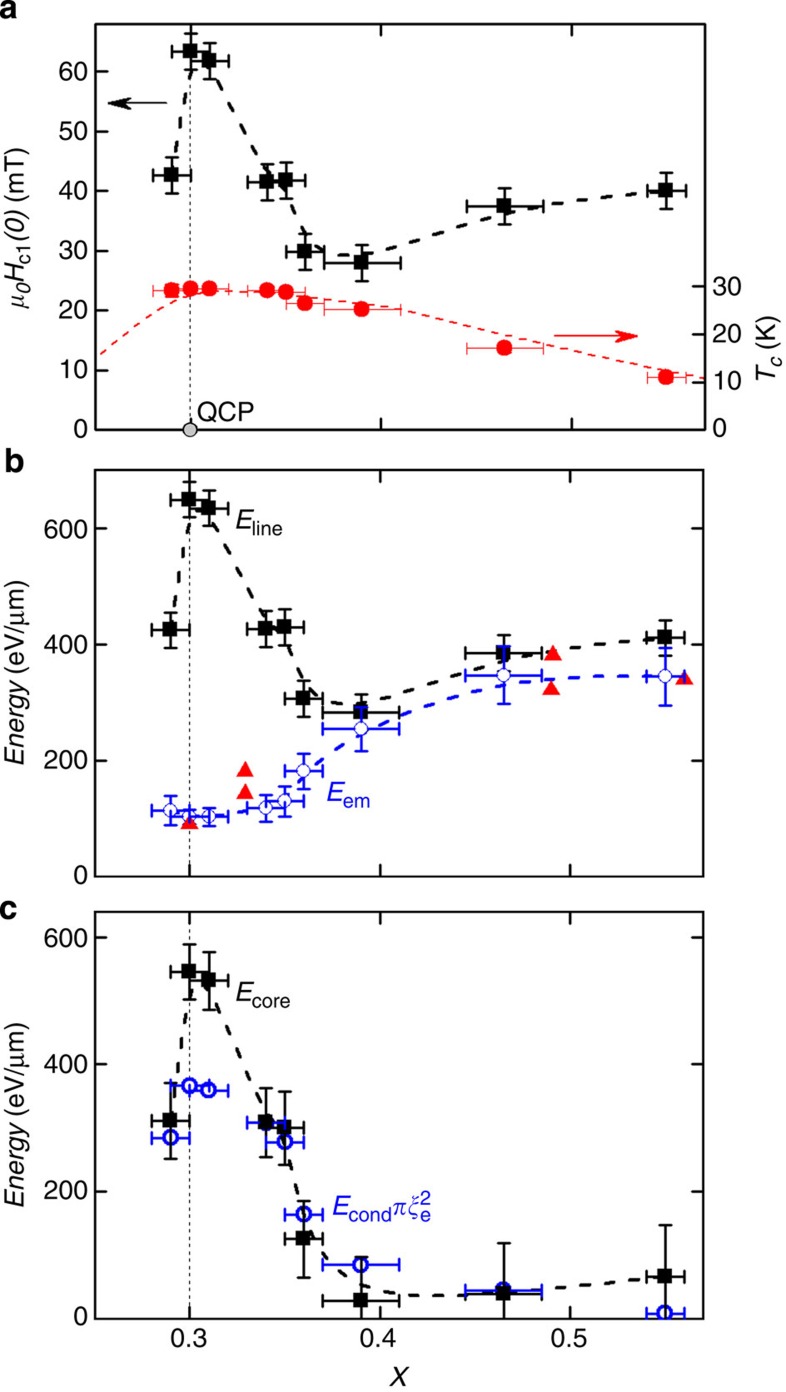
Concentration *x* dependence of lower critical field and associated energies for BaFe_2_(As_1−*x*_P_*x*_)_2_. (**a**) Lower critical field *H*_c1_ extrapolated to *T*=0 and *T*_c_. The location of the QCP is indicated. Error bars on *H*_c1_ represent the combination of uncertainties in extrapolating *H*_c1_(*T*) to *T*=0 and in the demagnetizing factor. Error bars on *x* are s.d. (**b**) Vortex line energy *E*_line_=*E*_em_+*E*_core_ at *T*=0 from the *H*_c1_(0) data and equations (4) and (3) shown as squares. The electromagnetic energy calculated using equation (4) and different estimates of *λ* are also shown. The triangles are direct measurements from ref. 9, and the circles are estimates derived by scaling the band-structure value of *λ* by the effective mass enhancement from specific heat 10. Error bars on *E*_em_ (circles) are calculated from the uncertainty in jump size in heat capacity at *T*_c_. (**c**) Vortex core energy *E*_core_=*E*_line_−*E*_em_ along with an alternative estimate derived from the specific heat condensation energy (*E*_cond_) and the effective vortex area (*πξ*_e_^2^). The uncertainties are calculated from a combination of those in the other panels. The dashed lines in all panels are guides to the eye.

The temperature dependence of *H*_c1_ is found to be linear in *T* at low temperature for all *x* (Fig. 3), which again is indicative of a lack of surface barriers that tend to become stronger at low temperature causing an upturn in *H*_c1_(*T*)^16^. Extrapolating this linear behaviour to zero temperature gives us *H*_c1_(0), which is plotted versus *x* in Fig. 4a. Surprisingly, instead of a dip in *H*_c1_(0) at the QCP predicted by equation (2) in conjunction with the observed behaviour of *λ*(*x*)^9^, there is instead a strong peak. To resolve this discrepancy we consider again the arguments leading to equation (2).

In general *H*_c1_ is determined from the vortex line energy *E*_line_, which is composed of two parts^17^,


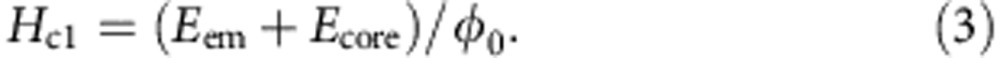


The first, *E*_em_ is the electromagnetic energy associated with the magnetic field and the screening currents, which in the high *κ* approximation is given by


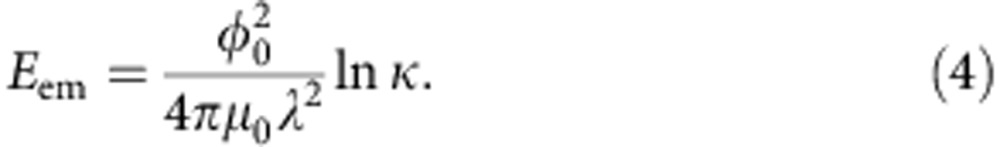


The second contribution arises from the energy associated with creating the normal vortex core *E*_core_. In high *κ* superconductors, *E*_core_ is usually almost negligible and is accounted for by the additional constant 0.5 in equation (2). However, in superconductors close to a QCP we argue this may not be the case.

In Fig. 4b,c we use equations (3) and (4) to determine *E*_em_ and *E*_core_. Away from the QCP, *E*_core_ is approximately zero and so the standard theory accounts for *H*_c1_(0) well. However, as the QCP is approached there is a substantial increase in *E*_core_ as determined from the corresponding increase in *H*_c1_. We can check this interpretation by making an independent estimate of the core energy from the condensation energy *E*_cond_, which we estimate from the experimentally measured specific heat (see Methods). The core energy is then 
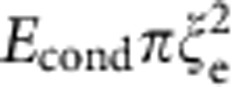
, where *ξ*_e_ is the effective core radius that may be estimated from the coherence length *ξ*_GL_ derived from *H*_c2_ measurements using equation (1). In Fig. 4, we see that 
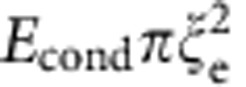
 has a similar dependence on *x* as *E*_core_ and is in approximate quantitative agreement if *ξ*_e_≅4.0*ξ*_GL_ for all *x*. Hence, this suggests that the observed anomalous increase in *H*_c1_ could be caused by the high energy needed to create a vortex core close to the QCP.

## Discussion

In principle, the relative lack of enhancement in *H*_c2_ close to the QCP could be caused by impurity or multiband effects, although we argue that neither are likely explanations. Impurities decrease *ξ*_GL_ and in the extreme dirty limit *H*_c2_∝m*T_c_/*ℓ*, where *ℓ* is the electron mean-free-path^11^. Hence, even in this limit we would expect *H*_c2_ to increase with m* although not as strongly as in the clean case. Impurities increase *H*_c2_ and as the residual resistance increases close to *x*=0.3 (ref. 7) we would actually expect a larger increase in *H*_c2_ than expected from clean-limit behaviour. dHvA measurements show that *ℓ*>>*ξ*_GL_ at least for the electron bands and for *x*>0.38, which suggest that, in fact, our samples are closer to the clean limit.

To discuss the effect of multiple Fermi surface sheets on *H*_c2_, we consider the results of Gurevich^18^ for two ellipsoidal Fermi surface sheets with strong interband pairing. This limit is probably the one most appropriate for BaFe_2_(As_1−*x*_P_*x*_)_2_(ref. 19). In this case for *H*||*c*, 
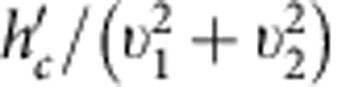
 were *υ*_1,2_ are the in-plane Fermi velocities on the two sheets. So if the velocity was strongly renormalized on one sheet only (*υ*_1_→0) then *H*_c2_ would be determined mostly by *υ*_2_ on the second sheet and hence would not increase with m* in accordance with our results. However, in this case the magnetic penetration depth *λ*, which will also be dominated by the Fermi surface sheet with the largest *υ*, would not show a peak at the QCP in disagreement with experiment^9^. In fact, the numerical agreement between the increase in m* with *x* as determined by *λ* or specific heat, which in contrast to *λ* is dominated by the low Fermi velocity sections, rather suggests that the renormalization is mostly uniform on all sheets^10^. In the opposite limit, appropriate to the prototypic multiband superconductor MgB_2_, where intraband pairing dominates over interband, *H*_c2_ will be determined by the band with the lowest *υ* (ref. 18) and again an increase in m* should be reflected in *H*_c2_. So these multiband effects cannot easily explain our results.

Another effect of multiband superconductivity is that it can modify the temperature dependence of *H*_c2_ such that it departs from the HW model. For example, in some iron-based superconductors a linear dependence of *H*_c2_(*T*) was found over a wide temperature range^20^. For BaFe_2_(As_1−*x*_P_*x*_)_2_, however, the coincidence between the HW extrapolation of the *H*_c2_ data close to *T*_c_ and the pulsed field measurement of *H*_irr_ for *T*≪*T*_c_ for all *x*, would appear to rule out any significant underestimation of *H*_c2_(0). In Supplementary Fig. 3 we show that *H*_irr_ for a sample with *x*=0.51 fits the HW theory for *H*_c2_(*T*) over the full temperature range. There is no reason why *H*_irr_ would underestimate *H*_c2_(0) by the same factor as the HW extrapolation. Even in cuprate superconductors where, unlike here, there is evidence for strong thermal fluctuation effects, *H*_irr_ has been shown to agree closely with *H*_c2_ in the low-temperature limit^21^. The magnitude of the discrepancy between the behaviour of *H*_c2_(0) and m* discussed above (see Fig. 2) also makes an explanation based on an experimental underestimate of *H*_c2_(0) implausible.

Another possibility is that in heavy fermion superconductors the mass enhancement is often reduced considerably at high fields and therefore m* could be reduced at fields comparable to *H*_c2_. In BaFe_2_(As_1−*x*_P_*x*_)_2_, however, a significantly enhanced mass in fields greater than *H*_c2_ can be inferred from the dHvA measurements^10^ and low temperature, high field, resistivity^22^. Although very close to the QCP the mass inferred from these measurements is slightly reduced from the values inferred from the zero field specific heat measurements^10^ this cannot account for the lack of enhancement of *H*_c2_ shown in Fig. 2.

Our results are similar to the behaviour observed in another quantum critical superconductor, CeRhIn_5_. Here the pressure tuned QCP manifests a large increase in the effective mass as measured by the dHvA effect and the low-temperature resistivity. *T*_c_ is maximal at the QCP but *H*_c2_ displays only a broad peak, inconsistent with the mass enhancement shown by the other probes^23^. We should note that in this system *H*_c2_ at low temperatures is Pauli limited. However, close to *T*_c_, *H*_c2_ is always orbitally limited and as neither *h*′ or *H*_c2_(0) are enhanced in BaFe_2_(As_1−*x*_P_*x*_)_2_ or CeRhIn_5_ (ref. 23), Pauli limiting can be ruled out as the explanation.

A comparison with the behaviour observed in cuprates is also interesting. Here two peaks in *H*_c2_(0) as a function of doping *p* in YBa_2_Cu_3_O_7−*δ*_ have been reported^21^, which approximately coincide with critical points where other evidence suggests that the Fermi surface reconstructs. Quantum oscillation measurements indicate that m* increases close to these points^24^, suggesting a direct link between *H*_c2_(0) and m* in the cuprates in contrast to our finding here for BaFe_2_(As_1−*x*_P_*x*_)_2_. However, by analysing the data in the same way as we have done here, it can be seen^25^ that *H*_c2_(0)^0.5^/*T*_c_ for YBa_2_Cu_3_O_7−*δ*_ is independent of *p* above *p*≅0.18 and falls for *p* below this value, reaching a minimum at *p*≅1/8. This suggests that at least the peak at higher *p* is driven by the increasing gap value rather than a peak in m*, in agreement with our results here, and that the minimum in *H*_c2_(0)^0.5^/*T*_c_ coincides with the doping where charge order is strongest at *p*≅1/8 (ref. 26).

The lack of enhancement of *H*_c2_(0) in all these systems suggests a fundamental failure of the theory. One possibility is that this may be driven by microscopic mixing of superconductivity and antiferromagnetism close to the QCP. In the vicinity of the QCP, antiferromagnetic order is expected to emerge near the vortex core region where the superconducting order parameter is suppressed^27^,^28^. Such a field-induced antiferromagnetic order has been observed experimentally in cuprates^29^,^30^. When the QCP lies beneath the superconducting dome, as in the case of BaFe_2_(As_1−*x*_P_*x*_)_2_ (refs 4, 9), antiferromagnetism and superconductivity can coexist on a microscopic level. In such a situation, as pointed out in ref. 28, the field-induced antiferromagnetism can extend outside the effective vortex core region where the superconducting order parameter is finite. Such an extended magnetic order is expected to lead to further suppression of the superconducting order parameter around vortices. This effect will enlarge the vortex core size, which in turn will suppress the upper critical field in agreement with our results. We would expect this effect to be a general feature of superconductivity close to an antiferromagnetic QCP, but perhaps not relevant to the behaviour close to *p*=0.18 in the cuprates.

To explain the *H*_c1_ results we postulate that the vortex core size is around four times larger than the estimates from *H*_c2_. This is in fact expected in cases of multiband superconductivity or superconductors with strong gap anisotropy. In MgB_2_ (refs 31, 32) and also in the anisotropic gap superconductor 2*H*-NbSe_2_ (ref. 33) the effective core size has been found to be around three times *ξ*_GL_, similar to that needed to explain the behaviour here. BaFe_2_(As_1−*x*_P_*x*_)_2_ is known to have a nodal gap structure^34^, which remains relatively constant across the superconducting dome^9^ and so we should expect the core size to be uniformly enhanced for all *x*. The peak in *H*_c1_(*x*) at the QCP is then, primarily caused by the fluctuation-driven enhancement in the normal-state energy, but the effect is magnified by the nodal gap structure of BaFe_2_(As_1−*x*_P_*x*_)_2_.

We expect the observed anomalous increase in *H*_c1_ to be a general feature of quantum critical superconductors as these materials often have nodal or strongly anisotropic superconducting gap structures and the increase in normal state energy is a general property close to a QCP. The relative lack of enhancement in *H*_c2_ also seems to be a general feature, which may be linked to a microscopic mixing of antiferromagnetism and superconductivity.

## Methods

### Sample growth and characterization

BaFe_2_(As_1−*x*_P_*x*_)_2_ samples were grown using a self-flux technique as described in ref. 7. Samples for this study were screened using specific heat and only samples with superconducting transition width <1 K were measured (see Supplementary Fig. 1). To determine the phosphorous concentration in the samples we carried out energy-dispersive X-ray analysis on several randomly chosen spots on each crystal (*H*_c1_ samples) or measured the *c* axis lattice parameter using X-ray diffraction (*H*_c2_ samples), which scales linearly with *x*. For some of the *H*_c2_ samples measured using high-field torque magnetometry the measured de Haas–van Alphen frequency was also used to determine *x* as described in ref. 10.

### Measurements of *H*_c2_

Close to *T*_c_ the upper critical field was determined using heat capacity. For this a thin film microcalorimeter was used^10^. We measured the superconducting transition at constant magnetic field up to 14 T (see Supplementary Fig. 2). The midpoint of the increase in *C* at the transition defines *T*_c_(*H*). At low temperatures (*T*≪*T*_c_) we used piezo-resistive microcantilevers to measure the magnetic torque in pulsed magnetic field and hence determine the irreversibility field *H*_irr_. The crystals used in the pulsed field study were the same as those used in ref. 10 for the de Haas–van Alphen effect (except samples for *x*≅0.3). By taking the difference between the torque in increasing and decreasing field we determined the point at which the superconducting hysteresis closes as *H*_irr_ (see Fig. 1b). For some compositions we measured *H*_irr_ in d.c. field over the full temperature range and found it to agree well with the HW model and also the low-temperature measurements in pulsed field on the same sample (Supplementary Fig. 3). Our heat capacity measurements of *H*_c2_ close to *T*_c_(*H*=0) are in good agreement with those of ref. 35.

### Measurements of *H*_c1_

The measurements of the field of first flux penetration *H*_p_ have been carried out using micro-Hall arrays. The Hall probes were made with either GaAs/AlGaAs heterostructures (carrier density *n*_s_=3.5 × 10^11^*cm*^−2^) or GaAs with a 1 μm thick silicon doped layer (concentration *n*_s_=1 × 10^16^*cm*^−3^). The latter had slightly lower sensitivity but proved more reliable at temperatures below 4 K. The measurements were carried out using a resistive magnet so that the remanent field during zero field cooling was as low as possible. The samples were warmed above *T*_c_ after each field sweep and then cooled at a constant rate to the desired temperature.

When strong surface pinning is present *H*_p_ may be pushed up significantly beyond *H*_c1_. In this case there will also be a significant difference between the critical field *H*_p_ measured at the edge and the centre of the sample (for example see ref. 15) and also a difference between the field where flux starts to enter the sample and the field at which it leaves. Some of our samples, also showing signs of inhomogeneity, such as wide superconducting transitions, showed this behaviour. An example is shown in Supplementary Fig. 4. In this sample the sensor at the edge shows first flux penetration at *H*_p_≈5 mT, whereas the value is ~3 times higher at the centre. For decreasing fields, the centre sensor shows a similar value to the edge sensor. All the samples reported in this paper showed insignificant difference between *H*_p_ at the centre and the edge and also for increasing and decreasing fields. Hence, we conclude that *H*_c1_ in our samples is not significantly increased by pinning.

As our samples are typically thin platelets, demagnetization effects need to be taken into account for measurement of *H*_c1_. Although an exact solution to the demagnetization problem is only possible for ellipsoids and infinite slabs, a good approximation for thin slabs has been obtained by Brandt^36^. Here *H*_c1_ is related to the measured *H*_p_, determined from *H* using


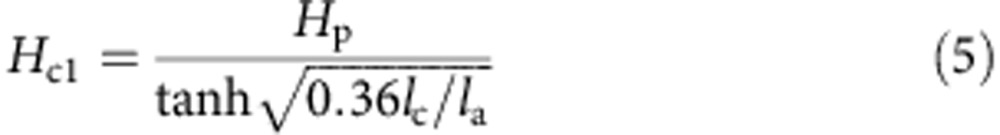


where *l*_c_ is the sample dimension along the field and *l*_a_ perpendicular to the field.

All samples in this study had *l*_c_≪*l*_a_. To ensure that the determination of the effective field is independent of the specific dimension we have carried out multiple measurements on a single sample cleaved to give multiple ratios of *l*_c_/*l*_a_. The results of this study (Supplementary Fig. 5) show that *H*_c1_ determined by this method are independent of the aspect ratio of the sample. Furthermore, the samples used all had similar *l*_c_/*l*_a_ ratios (see Supplementary Table 1), and so any correction would not give any systematic errors as a function of *x*.

### Calculation of condensation energy

The condensation energy can be calculated from the specific heat using the relation


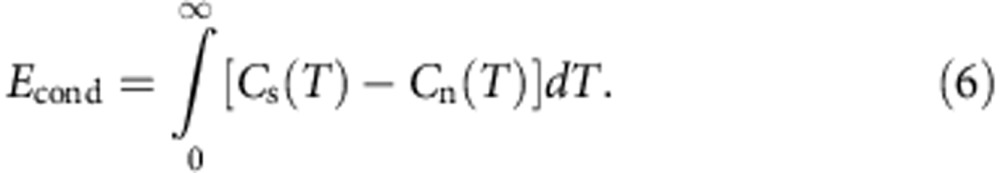


To calculate this, we first measured a sample of BaFe_2_(As_1−*x*_P_*x*_)_2_ with *x*=0.47, using a relaxation technique in zero field and *μ*_0_*H*=14 T, which is sufficient at this doping to completely suppress superconductivity and thus reach the normal state. We used this 14 T data to determine the phonon heat capacity and we then subtract this from the zero field data to give the electron specific heat of the sample. We then fitted this data to a phenomenological nodal gap, alpha model (with variable zero temperature gap) similar to that described in ref. 37 (see Supplementary Fig. 6). We then integrated this fit function using equation (6) to give *E*_cond_ for this value of *x*. For lower values of *x* (higher *T*_c_) the available fields were insufficient to suppress superconductivity over the full range of temperature, so we assumed that the shape of the heat capacity curve does not change appreciably with *x* but rather just scales with *T*_c_ and the jump height at *T*_c_. This is implicitly assuming that the superconducting gap structure does not change appreciably with *x*, which is supported by magnetic penetration depth *λ* measurements which show that normalized temperature dependence *λ*(*T*)/*λ*(0) is relatively independent of *x*^9^. With this assumption we can then calculate


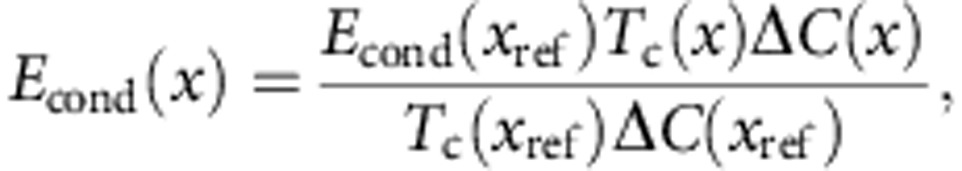


where *x*_ref_=0.47.

## Author contributions

A.C. and C. Putzke. conceived the experiment. C. Putzke performed the high-field torque measurements (with D.V., C. Proust and S.B.) and the Hall probe measurements. P.W. and L.M. performed heat capacity measurements. The Hall probe arrays were fabricated by J.D.F., P.S., H.E.B and D.A.R. Samples were grown and characterized by S.K., Y. Mizukami, T.S. and Y.Matsuda. The manuscript was written by A.C. with input from C. Putzke, C. Proust, P.W., L.M., J.D.F, T.S and Y. Matsuda.

## Additional information

**How to cite this article:** Putzke, C. *et al.* Anomalous critical fields in quantum critical superconductors. *Nat. Commun.* 5:5679 doi: 10.1038/ncomms6679 (2014).

## Supplementary Material

Supplementary Information Supplementary Figures 1-6 and Supplementary Table 1
