# Supplementary material for: Anomalous critical fields in quantum critical superconductors
Source: Nat Commun. 2014 Dec 5;5:5679. doi: 10.1038/ncomms6679 (PMC4268691; doi:10.1038/ncomms6679)
Supplement: Supplementary Information — Supplementary Figures 1-6 and Supplementary Table 1 [file ncomms6679-s1.pdf]

SUPPLEMENTARY INFORMATION

Supplementary Figure 1

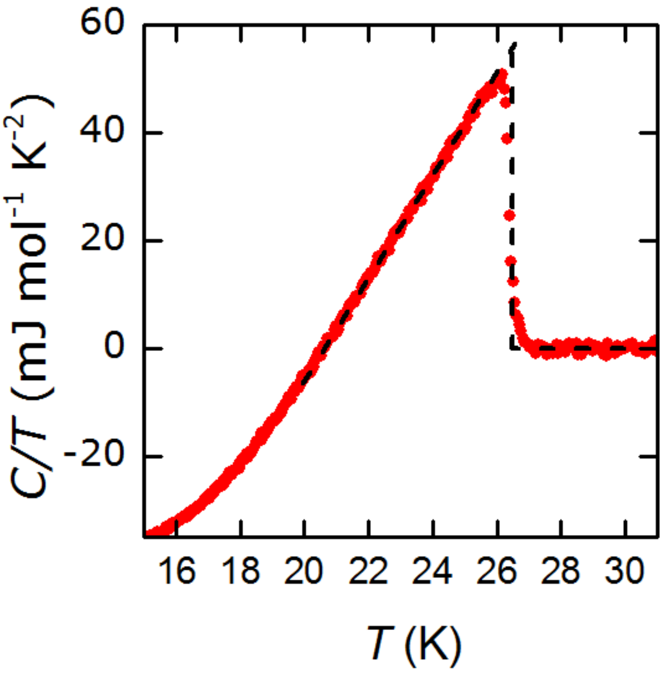

Supplementary Figure 1 Heat capacity of a sample with $x = 0.38$ with the normal state heat capacity subtracted. The dashed line is the behaviour expected for a mean-field like jump.

Supplementary Figure 2

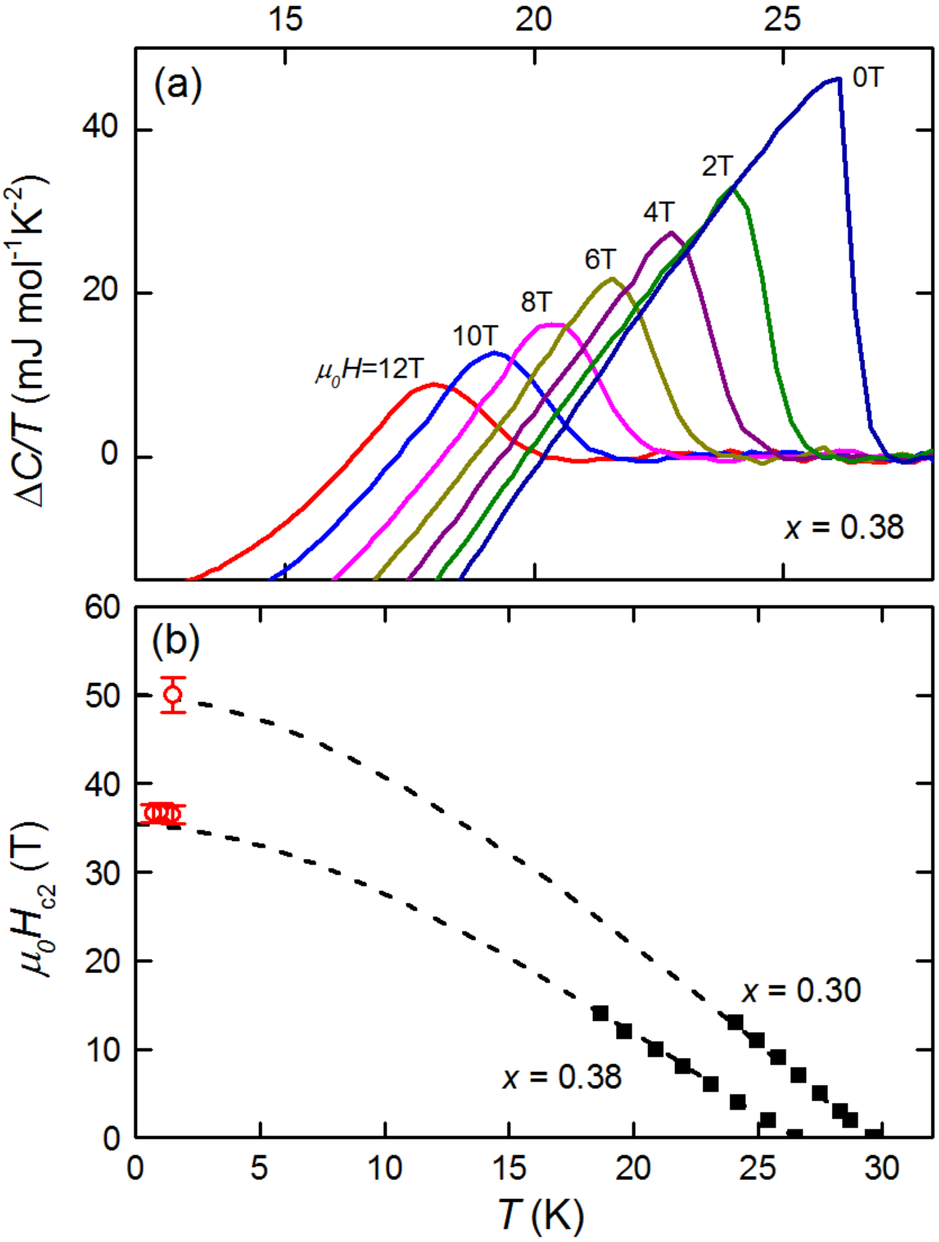

Supplementary Figure 2 Determination of H_{c2}. (a) Heat capacity jump of a sample with $x = 0.38$ at various magnetic fields up to 12 T. A polynomial fit was used to subtract the normal state specific heat. Panel (b) shows H_{c2} versus temperature for the $x = 0.38$ sample shown in (a) determined from the midpoint of the jump in the heat capacity and similar data for a sample with $x = 0.30$. The open circles are values of H_{irr} as determined by torque magnetometry in pulsed field on two other samples with the same values of x and T_{c}. The dashed lines are fits to the clean limit HW model [14] for $H_{\mathrm{c2}}(T)$.

Supplementary Figure 3

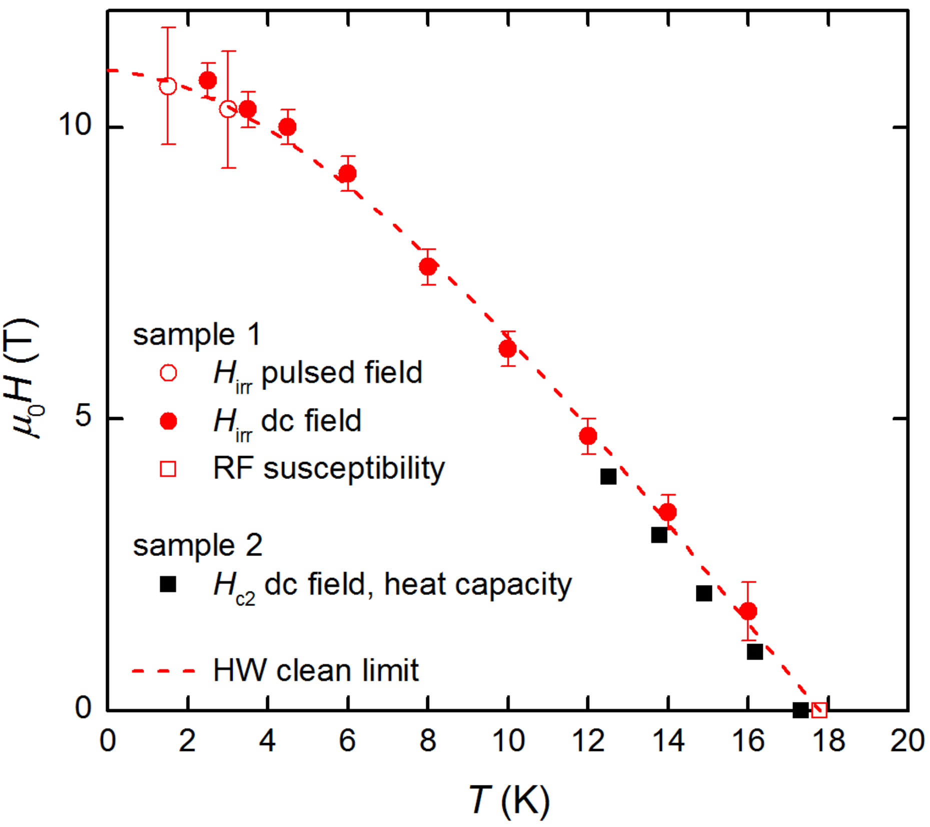

Supplementary Figure 3 Irreversibility field H_{irr}, as determined by torque measurements, versus temperature for a sample with $x = 0.51 \pm 0.02$ over the full temperature range. The solid circles are measurements in dc field and the open circles are in pulsed field (same sample). The T_{c} in zero field of this sample was determined by very low field radio-frequency susceptibility measurements. The solid squares are measurements of H_{c2} determined from the midpoint of the jump in the specific heat of a second sample with almost the same x (T_{c} of this sample is 0.4 K lower than the torque sample in zero field, and x is the same within error by EDX). The dashed line is a fit to the clean limit HW model [14] for $H_{\mathrm{c2}}(T)$.

Supplementary Figure 4

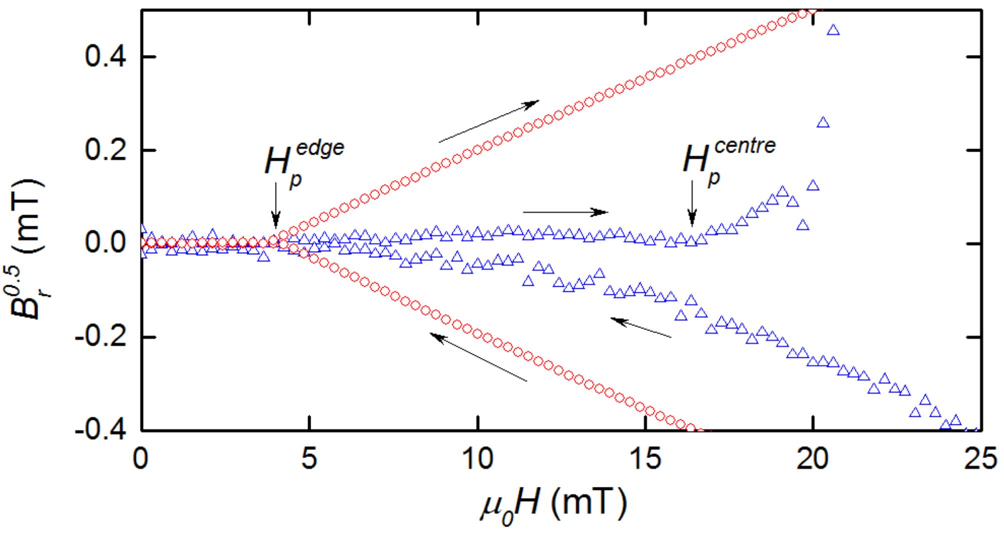

Supplementary Figure 4 Remnant field B_{r} of a sample with $x = 0.30$ that showed a wide superconducting transition of $\Delta T_{\mathrm{c}} > 1.5\,\mathrm{K}$. Two sensors at the edge (circles) and at the centre of the sample (triangles) are shown.

Supplementary Figure 5

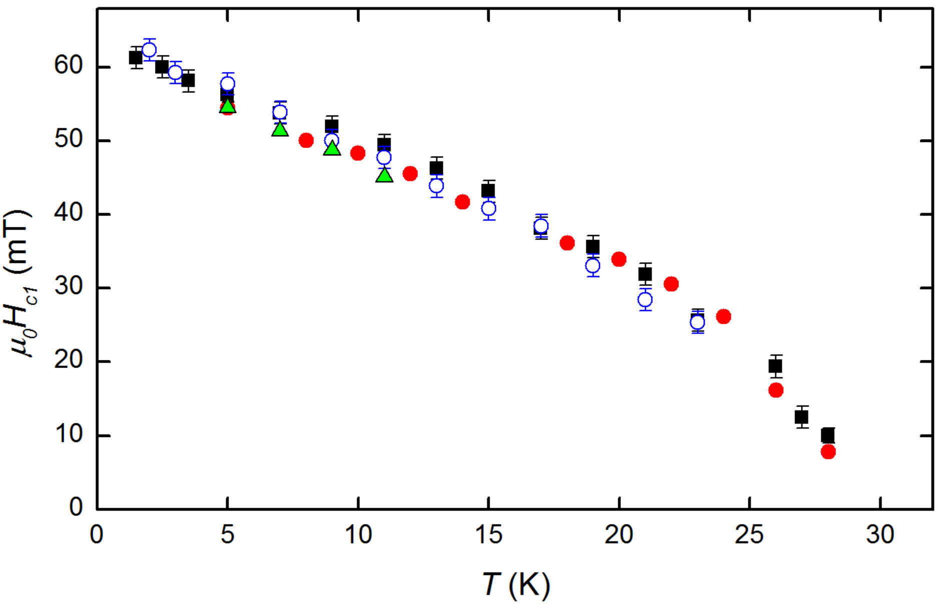

Supplementary Figure 5 $H_{\mathrm{c1}}(T)$ for samples C24 and C2a with $x = 0.3$ and $x = 0.31$ respectively. Sample C24 has been cleaved repeatedly to produce samples with different $l_{\mathrm{c}}/l_{\mathrm{a}}$ ratios: 0.05 (C24,open circles), 0.08(C24,squares), 0.09 (C2a,filled circles) and 0.11 (C24,triangles), in order to test the accuracy of the demagnetising factor determination.

Supplementary Figure 6

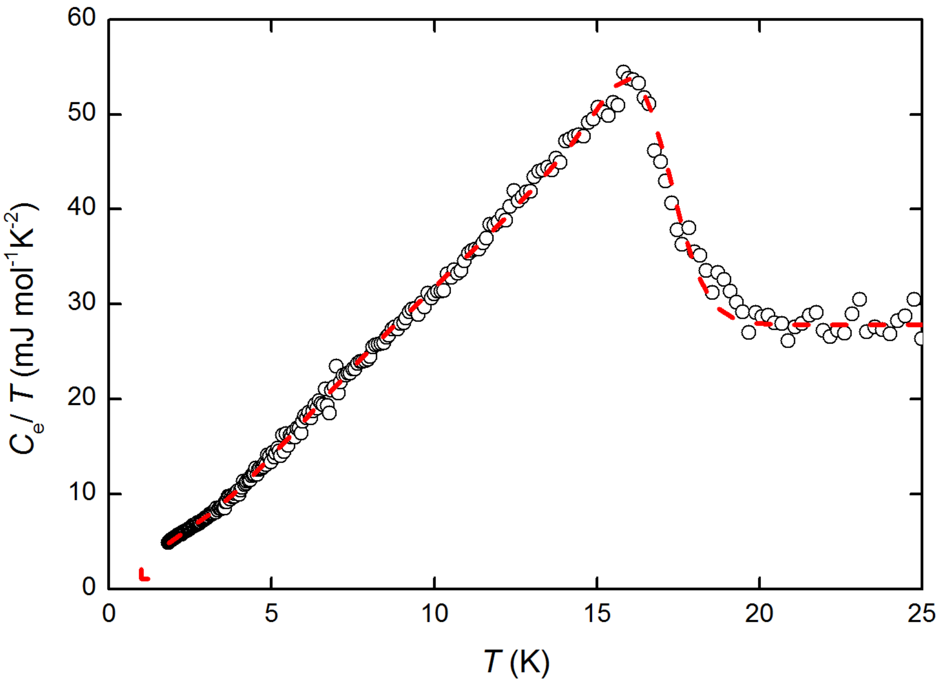

Supplementary Figure 6 Heat capacity of a $x = 0.47$ sample with the phonon contribution subtracted. The phonon contribution was determined directly by using a high field (14,T) to suppress the superconductivity. The dashed line is a fit to the data using a nodal gap alpha model similar to Ref. 36. The model has been convoluted with a Gaussian distribution to model the spread of T_c in the sample.

Supplementary Table 1

Sample	x	Δx	l_c (μm)	l_a (μm)
C19	0.29	0.01	11	149
C24a	0.30	0.01	27	360
C24b	0.30	0.01	18	360
C24c	0.30	0.01	18	170
C2a	0.31	0.01	28	300
C21	0.34	0.01	20	255
0p3B	0.35	0.01	48	115
C1	0.36	0.01	35	269
C7a	0.39	0.02	17	260
C9	0.47	0.02	40	300
0p6a	0.55	0.01	48	240

Supplementary Table 1 List of dimension of the samples used for the H_{c1} measurements.
